# A20 in dendritic cells restrains intestinal anti-bacterial peptide expression and preserves commensal homeostasis

**DOI:** 10.1371/journal.pone.0218999

**Published:** 2019-07-11

**Authors:** Alice Talpin, Michael G. Kattah, Rommel Advincula, Douglas Fadrosh, Kole Lynch, Brandon LaMere, Kei E. Fujimura, Nabeetha A. Nagalingam, Barbara A. Malynn, Susan V. Lynch, Averil Ma

**Affiliations:** Department of Medicine, University of California, San Francisco, San Francisco, CA, United States of America; Kurume University School of Medicine, JAPAN

## Abstract

Microbial dysbiosis commonly occurs in patients with inflammatory bowel diseases (IBD). Exogenous causes of dysbiosis such as antibiotics and diet are well described, but host derived causes are understudied. A20 is a potent regulator of signals triggered by microbial pattern molecules, and A20 regulates susceptibility to intestinal inflammation in mice and in humans. We now report that mice lacking A20 expression in dendritic cells, A20^FL/FL^ CD11c-Cre mice (or A20^dDC^ mice), spontaneously develop colitogenic intestinal dysbiosis that is evident upon weaning and precedes the onset of colitis. Intestines from A20^dDC^ mice express increased amounts of Reg3β and Reg3γ, but not Ang4. A20 deficient DCs promote gut microbiota perturbation in the absence of adaptive lymphocytes. Moreover, A20 deficient DCs directly induce expression of Reg3β and Reg3γ but not Ang 4 in normal intestinal epithelial cell enteroid cultures in the absence of other cell types. These findings reveal a pathophysiological pathway in which defective expression of an IBD susceptibility gene in DCs drives aberrant expression of anti-bacterial peptides and luminal dysbiosis that in turn confers host susceptibility to intestinal inflammation.

## Introduction

Symbiosis between commensal microbes and host immune cells in the intestine involves bidirectional secretion of molecules that provide homeostatic signals [1,2]. Perturbations of this cross talk can lead to both microbiome disturbance and host disease. Intestinal dysbiosis has been linked to a variety of inflammatory conditions in human patients and in mice [3]. While exogenous perturbations such as antibiotics and dietary changes are known to perturb luminal microbiomes^3^, altered functions of host cells may also drive microbial dysfunction [4–7].

TNFAIP3, which encodes the A20 protein, is linked genetically and epigenetically to inflammatory bowel disease (IBD). Moreover, patients bearing mono-allelic mutations in A20 coding sequences develop skin, mucosal and intestinal inflammation at young ages [8]. Hence, the clinical ties between A20 and IBD are extensive and compelling. A20 is expressed in multiple cell types and is a potent regulator of responses to microbial ligands, including TLR and NOD2 triggered signals [9–12]. Dendritic cells (DCs) are particularly well endowed with microbial sensing proteins and are positioned at the intersection of innate and adaptive immune responses. Distinct subsets of intestinal DCs utilize A20 to restrict microbial signals [13, 14], and mice bearing A20 deficient DCs spontaneously develop colitis and spondyloarthritis [13] or autoimmunity [15]. As A20 deficient DCs exert potent physiological influences upon intestinal immune homeostasis, we investigated whether these cells might regulate the composition of intestinal microbial communities.

## Results and discussion

**Fig 1:**
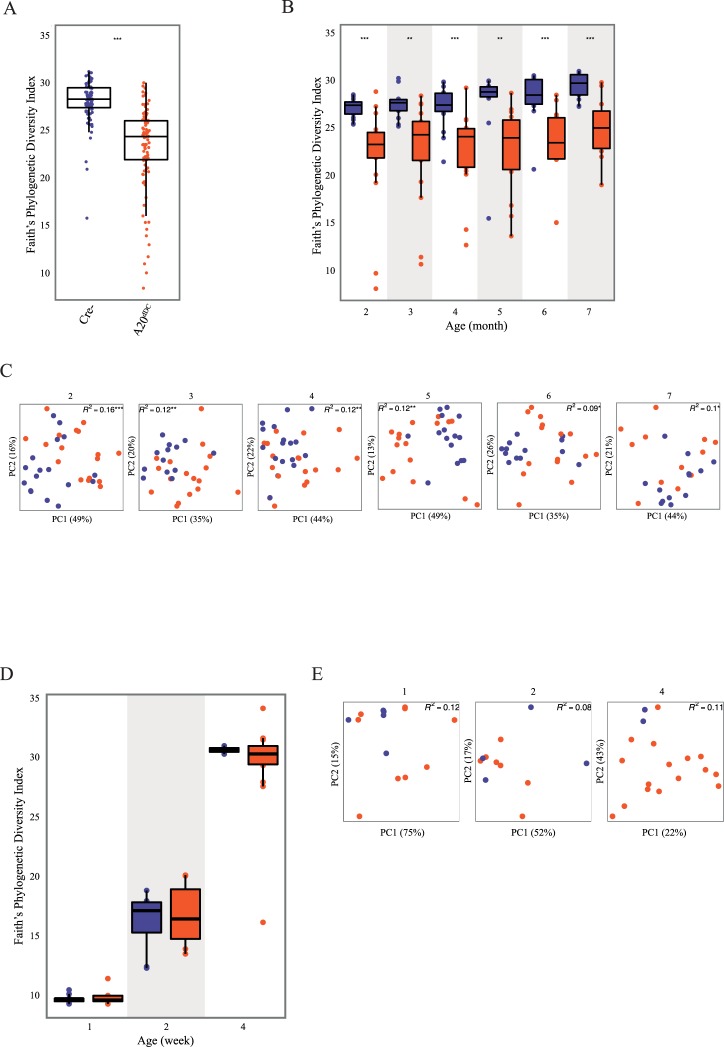
A20 expression in dendritic cells preserves microbial homeostasis. (A) Between-group and (B) cross-sectional analysis of Faith’s phylogenetic diversity of fecal samples collected longitudinally from 2 to 7 month old A20^dDC^ mice (red circles) and A20^FL/FL^ (Cre-) control mice (blue circles). (C) β-diversity analyses of microbial communities from 2 to 7 month-old A20^dDC^ mice (red circles) and A20^FL/FL^ (Cre-) mice (blue circles); *** = p<0.001, ** = p<0.01, * = p <0.05. (D) Faith’s Phylogenetic diversity and (E) β-diversity analyses of fecal microbiota from 1 week, 2 week and 4 week-old co-housed A20^dDC^ mice (red) and control A20^FL/FL^ (Cre-) mice (blue). No statistically significantly differences in microbiota from 1, 2, or 4 week-old pups were observed.

We previously observed that mice lacking A20 expression in DCs (A20^FL/FL^ CD11c-Cre mice, or as termed herein, A20^dDC^ mice) spontaneously develop intestinal inflammation after 4 month of age [13]. Given the importance of DCs in regulating intestinal immune homeostasis, we hypothesized that aberrant DC function due to A20 deficiency might perturb luminal microbiota. To test this hypothesis, we explored the temporal evolution of microbial dysbiosis in these mice using 16S ribosomal RNA (16S rRNA) sequencing of samples collected between 2–7 months of life. These data indicated that reduced alpha diversity (Fig 1A and 1B) and distinct microbiota composition (Fig 1C) are consistent features of A20^dDC^ mice compared to control A20^FL/FL^ (Cre^-^) mice over the period of observation (Fig 1A–1C). A between-group comparison of taxon relative abundance (S1 Fig) across these repeated measures identified sporadic and age-dependent genus and taxon enrichments (S2 Fig). A large group of organisms including taxa belonging primarily to the *Bacteroides*, *Parabacteroides* and *Desulfovibrio* were consistently enriched throughout adulthood in A20^dDC^ mice compared with control animals (S1 Table). These data indicate a strong association between A20 ablation in DCs and distinct gut microbiota successional trajectories into adulthood, and suggest that A20 deficient DCs perturb luminal microbes before driving histological evidence of colitis.

Intestinal microbes are shared between littermate pups and their mothers prior to weaning. Emerging evidence from human studies indicates that early life gut microbiota dysbiosis precedes the development of chronic inflammatory disease in childhood [16–18]. We thus asked whether ablation of A20 from DCs plays a role in modulating the gut microbiota prior to weaning. We profiled the fecal bacterial microbiota of co-housed 1, 2 and 4 week old A20^dDC^ and A20^FL/FL^ (Cre^–^) littermate pups from the same A20^FL/FL^ (Cre^–^) dams. While a small number of taxa were modestly enriched in 2 or 4 week old A20^dDC^ pups compared with control (Cre-) littermates, these experiments revealed no significant differences in bacterial microbiota alpha or beta-diversity in 1, 2 or 4 week old A20^dDC^ mice compared to controls (Fig 1D and 1E and S1 Fig) (S2 Table). Collectively these data suggest taxonomic but not global differences in the gut microbiota of A20^dDC^ mice versus control mice prior to weaning, which probably reflects the early-life selective pressure of maternal breast milk on these communities at this developmental stage [19]. Differences in microbiota composition became more pronounced in A20^dDC^ pups between 4 and 8 weeks of age, as pups transition to eating solid food. Hence, microbiota perturbation evolves in A20^dDC^ pups as their microbiomes diversify in response to complex diets (S1 Fig).

**Fig 2:**
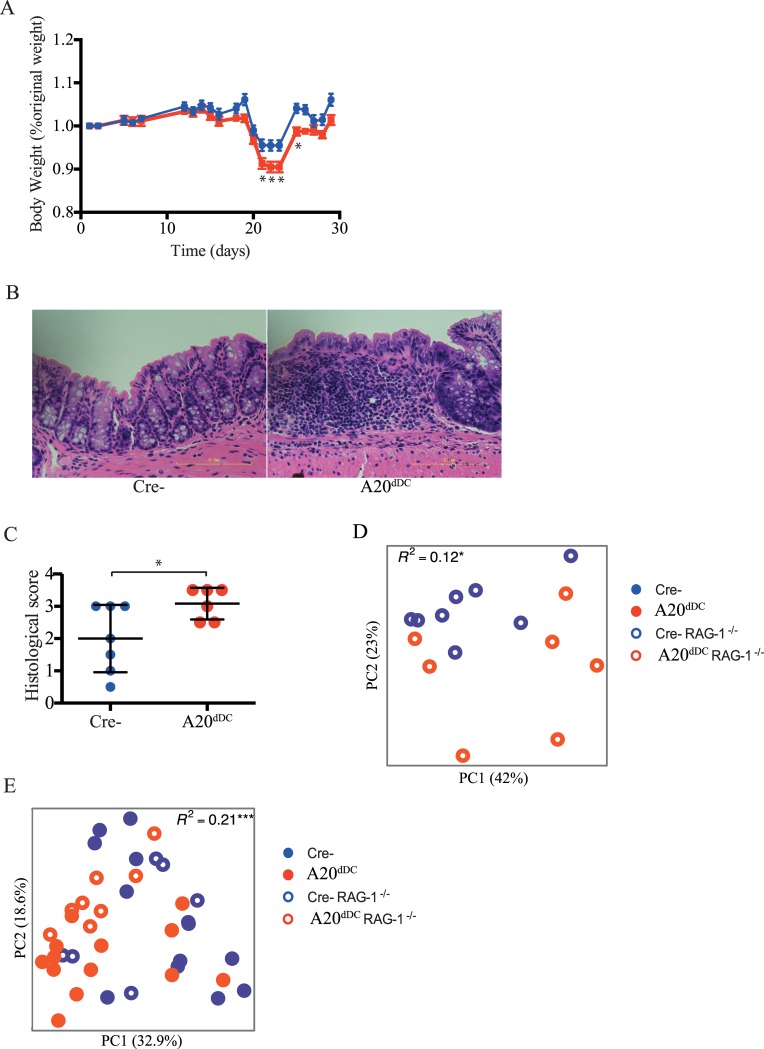
Dysbiotic microbes from A20^Fl/Fl^ CD11c-Cre mice confer susceptibility to intestinal inflammation. (A) Body weights of C57BL/6J wild type (WT) mice that received fecal microbiota transfer from either A20^dDC^ mice (red, n = 8) or A20^FL/FL^ (Cre-) control mice (blue, n = 8) and were subsequently treated with DSS for 5 days. (B) Representative images of H&E stained colonic tissues from mice in (A). Original magnification x 40. (C) Histological scores from colonic tissues shown in (A and B) (n = 6 mice per group). Data are representative of two independent experiments. (D) β-diversity analyses of fecal microbiota communities from intact 6 month-old A20^dDC^ RAG-1^-/-^ mice (red open circles, n = 7) and control A20^FL/FL^ (Cre-) RAG-1^-/-^ mice (blue open circles, n = 8 mice); * = p <0.05. (E) β-diversity analyses of fecal microbiota from A20^dDC^ mice (filled red circles, n = 13), A20^FL/FL^ (Cre-) mice (filled blue circles, n = 13), A20^dDC^ mice RAG-1^-/-^ (open red circles, n = 6), and A20^FL/FL^ (Cre-) RAG-1^-/-^ mice (open blue circles, n = 8) *** = p <0.001.

The data above indicate that germline encoded genetic changes affecting host intestinal myeloid cells drive commensal microbiota perturbation soon after weaning. Such perturbations could in turn amplify host susceptibility to inflammation. To determine whether microbiota perturbation in A20^dDC^ mice contributes to intestinal inflammation in normal mice, we harvested stool pellets from 7 month old A20^dDC^ mice or control mice, gavaged bacteria from these fresh stool pellets into normal syngeneic C57BL/6J mice, and then tested the susceptibility of the recipient mice to dextran sulfate sodium (DSS). These studies revealed that mice harboring A20^dDC^ mouse derived gut microbiomes exhibited greater weight loss and inflammatory damage (Fig 2A and 2B). Hence, intestinal dysbiosis triggered by A20 deficient DCs is colitogenic.

A20 deficient DCs perturb T and B cell tolerance [13, 15], and lymphocyte derived cytokines and anti-commensal IgA antibodies can modulate luminal microbiota composition. To determine whether adaptive lymphocytes are necessary for the observed dysbiosis in A20^dDC^ animals, we interbred these mice with RAG-1^-/-^ mice and compared the bacterial communities present in fecal samples from 6 month-old A20^dDC^ RAG-1^-/-^ compound mutant mice with control A20^FL/FL^ (Cre^-^) RAG-1^-/-^ mice. These studies surprisingly revealed that luminal bacteria in A20^dDC^ RAG-1^-/-^ mice diverged significantly from A20 competent RAG-1^-/-^ mice (Fig 2C). In addition, four way analyses of 16S rRNA data from A20^dDC^ mice, A20^FL/FL^ (Cre^-^) mice, A20^dDC^ RAG-1^-/-^ mice, and A20^FL/FL^ (Cre^-^) RAG-1^-/-^ mice revealed that bacterial taxa that were overrepresented in lymphocyte deficient A20^dDC^ RAG-1^-/-^ mice relative to A20^FL/FL^ (Cre^-^) RAG-1^-/-^ mice resembled those overrepresented in lymphocyte replete A20^dDC^ mice relative to A20^FL/FL^ (Cre^-^) mice (Fig 2D) (S3 Table). Together these data indicate that A20 deficient DCs can perturb microbial homeostasis in a lymphocyte independent fashion.

**Fig 3:**
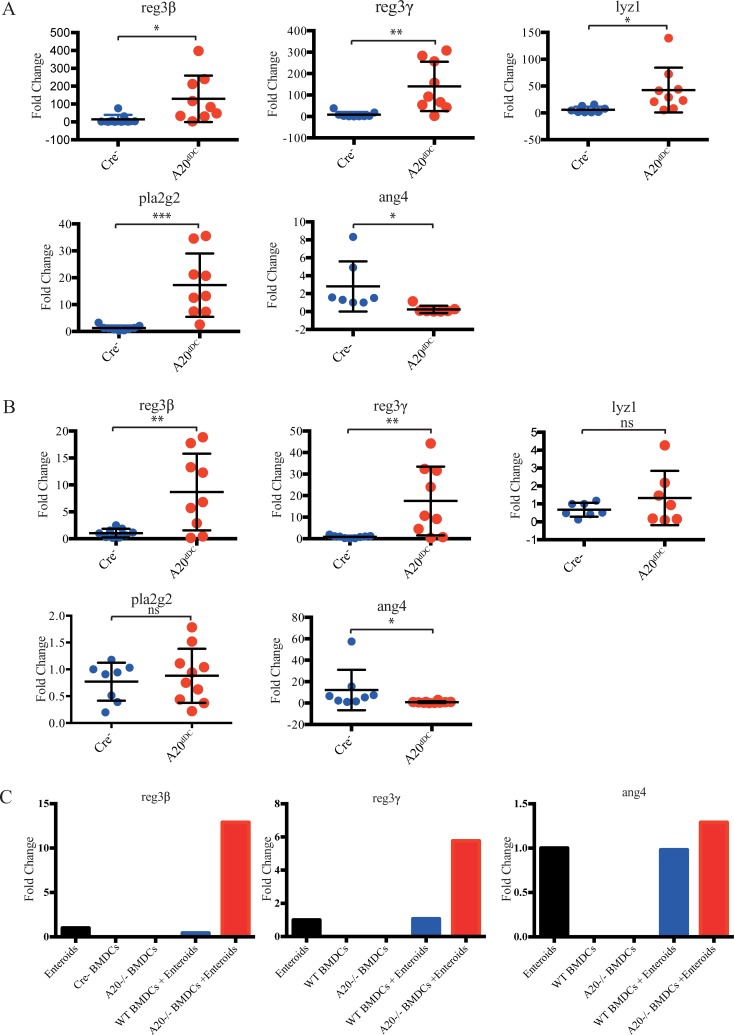
A20 deficient DCs increase transcription of anti-microbial defensins. (A) qPCR analyses of expression of the indicated anti-microbial defensins in proximate colons of A20^dDC^ mice (red, n = 10) and control A20^FL/FL^ (Cre-) mice (blue, n = 8). (B) qPCR analyses of expression of the indicated anti-microbial defensins in ileal tissues of A20^dDC^ mice (n = 9) and control A20^FL/FL^ (Cre-) mice (n = 9). Values are expressed as fold changes normalized to *hprt* expression, and represent means +/- SEM. * = p<0.05, ** = p<0.01, *** = p<0.001 by T-test. (C) qPCR analyses of expression of the indicated anti-microbial defensins from DC—IEC enteroid co-cultures. Bone marrow derived DCs that were either A20 deficient or A20 competent were co-cultured with WT IEC enteroids for 48 hours, after which co-cultures were harvested for qPCR analyses. Note that A20 deficient DCs cause greater IEC expression of selected defensins. Values are expressed as fold changes normalized to *hprt* expression, and represent means +/- SEM. * = p<0.05, ** = p<0.01, *** = p<0.001 by T-test. NB: Data are representative of 3 independent experiments.

Intestinal DCs elaborate a number of factors that could activate epithelial cells as well as other immune cells. Intestinal epithelial cell derived defensins and other anti-bacterial peptides are particularly well positioned to modulate intestinal bacterial communities. We thus analyzed intestinal expression of these molecules from A20^dDC^ and control mice via qPCR. These studies showed that intestines from A20^dDC^ mice expressed higher levels of the anti-microbial molecules Reg3β, Reg3γ, lysozyme, and Pla2g2, but not Ang4 when compared with WT mice in ileal or proximal colon tissues (Fig 3A and 3B). These molecules are selectively expressed by small intestinal Paneth cells and a subset of crypt epithelial cells in large intestines. Reg3β and Reg3γ are small proteins that are bactericidal for gram-positive bacteria. Pla2g2 is a phospholipase that kills bacteria by hydrolyzing bacterial membrane phospholipids. Ang4 utilizes RNAse activity to inhibit growth of both gram negative and positive bacteria [20–22]. These results suggest that A20 dependent DC functions limit anti-bacterial peptide expression *in vivo*. Moreover, exaggerated expression of these peptides in intestines of A20^dDC^ mice provide a mechanism by which A20 deficient DCs perturb luminal microbial homeostasis.

The data above indicate that A20 deficient DCs enhance intestinal expression of anti-bacterial peptides *in vivo*. These DCs could influence IEC expression of these molecules via a number of direct and/or indirect mechanisms. We thus asked whether A20 deficient DCs could directly regulate IEC functions. We prepared primary intestinal enteroid cultures from small intestines of normal mice, co-cultured these enteroids with A20 competent or A20 deficient BMDCs, and measured expression of anti-bacterial peptides by qPCR. These studies revealed that enteroids expressed higher levels of Reg3β and Reg3γ when co-cultured with A20 deficient DCs than when cultured with wild type DCs (Fig 3C). This finding indicates that DCs can directly regulate IEC functions in the absence of other intestinal cell types (e.g., adaptive lymphocytes, or innate immune cells).

In summary, this study reveals a new role for intestinal DCs in regulating intestinal microbial homeostasis. Gut dysbiosis in A20^dDC^ mice occurs with the introduction of solid food and confers susceptibility to intestinal inflammation in normal mice. A20 deficient DCs perturb microbial homeostasis in the complete absence of adaptive lymphocytes, and A20 deficient DCs can directly drive IEC expression of selected anti-bacterial peptides. Hence, in addition to prior studies showing that A20 expression in DCs preserves T cell tolerance [13], A20 expression in DCs also regulates IEC function. The current studies provide a second general pathway by which A20 expression in DCs preserves intestinal homeostasis.

## Material and methods

### Mice

A20^FL/FL^ CD11c-Cre mice, or A20^dDC^ mice, were generated in our laboratory and have been described (GA Hammer et al. 2011). C57BL/6J (stock no. 000664) and B6.129S7-Rag1^tm1Mom^/J (Rag1^−/−^, stock no. 002216) mice were purchased from Jackson Laboratories. All animal research was approved by University of California San Francisco (UCSF) Office of Ethics and Compliance under protocol # AN144847-03C.

### Fecal microbial transplant (FMT) and DSS colitis

Stool pellets were collected from 7 month old A20^dDC^ mice (n = 4) or A20^FL/FL^ (Cre^-^) control (n = 6) mice, pooled and homogenized in phosphate-buffered saline (PBS) at a ratio of 1g feces to 5ml of PBS. After vortexing vigorously, the fecal slurry was passed through a 70μm strainer and 100μL delivered to C57BL/6J recipient mice by oral gavage every 3 days for 15 days total. DSS-induced colitis treatment was started the day after the last fecal transfer. Mice were administered 3.5% dextran sulfate sodium (DSS) (MP Biomedicals) in drinking water (w/v) for 5 days followed by regular water. Mice were assessed daily for diarrhea and body weight.

### Dendritic cell–IEC enteroid co-culture

Bone marrow was obtained from femurs and tibias from A20^FL/FL^ ER/Rosa26-Cre mice femurs and control A20^FL/FL^ (Cre-) femurs, and cultured in DMEM containing 10% FCS and 10% GM-CSF. Media was changed every 3 days and supplemented with GM-CSF. 4-hydroxytamoxifen (4-OH-T) was added during the last 48 hours of the culture to delete A20 (100 nM; Sigma Aldrich). Purity of BMDCs after 9 days of culture was confirmed by CD11c and MHC II expression. Enteroid cultures were derived from intestinal crypts and cultured as previously described [23]. Confluent WT enteroids were resuspended in 50ul of matrigel (Corning) and co-cultured with 0.5 x 10^6^ BMDCs from A20^FL/FL^ ER/Rosa26-Cre mice or control A20^FL/FL^ (Cre-) mice. After 36 hrs of DC-IEC co-culture, all cells were harvested and total RNA was extracted.

### RNA extraction and quantitative RT-PCR

Total RNA was extracted from proximal ileum (1 cm proximal to the cecum) small intestine or proximal colon tissues with the RNeasy Mini Kit (Qiagen). Enteroid cultures were resuspended in Cell Recovery Solution (Corning) supplemented with 10 μM Y-27632 (Calbiochem) and incubated for 15 min on ice. Total RNA was isolated using the PicoPure RNA Isolation Kit (Thermo Fisher Scientific) according to the manufacturer instructions with on-column DNase I digestion (Qiagen). 500 ng total RNA was used for cDNA synthesis using the High Capacity RNA-to-cDNA kit (Thermo Fisher Scientific). qPCR was performed using the following mouse TaqMan probes: REG3B (Mm00440616_g1), REG3G (Mm00441127_m1), ANG4 (Mm03647554_g1), PLA2G2 (Mm00448160_m1), LYZ1 (Mm00657323_m1) and HPRT1 (Mm00446968_m1), and the TaqMan Universal Master Mix II with UNG on the QuantStudio 6 (Thermo Fisher Scientific). For *in vivo* intestinal experiments, relative gene abundance from A20^dDC^ containing samples was normalized to the mean expression of the housekeeping gene *hprt* and then compared to control A20^FL/FL^ (Cre^−^) samples. For *ex vivo* DC-enteroid co-culture experiments, relative gene abundance was normalized to the mean expression of the housekeeping gene *hprt* and then compared to enteroids alone as a reference. 2^−ΔΔCt^ was calculated. All samples were run in duplicate. Data were analyzed by using QuantStudio RealTime PCR Software (Thermo Fisher Scientific).

### Statistical analyses

Statistical analysis was performed with GraphPad Prism 6 (GraphPad Software). Comparisons between two groups were performed by two-tailed unpaired Student’s *t* test. P < 0.05 was used as the threshold for statistical significance.

### Fecal specimen processing and DNA extraction

Individual murine fecal samples were placed into lysing matrix E (LME) tubes pre-aliquoted with 500 of hexadecyltrimethylammonium bromide (CTAB) DNA extraction buffer and incubated at 65°C for 15 minutes. An equal volume of phenol:chloroform:isoamyl alcohol (25:24:1) was added to each tube and samples were homogenized in a Fast Prep-24 homogenizer at 5.5 m/s for 30 seconds. Tubes were centrifuged for 5 minutes at 16,000 x g and the aqueous phase was transferred to individual wells of a deep-well 96-well plate. An additional 500 μl of CTAB buffer was added to the LME tubes, the previous steps were repeated, and the aqueous phases were combined. An equal volume of chloroform was mixed with each sample, followed by centrifugation at 3000 x g for 10 minutes to remove excess phenol. The aqueous phase (600 μl) was transferred to a deep-well 96-well plate, combined with 2 volume-equivalents of polyethylene glycol (PEG) and stored overnight at 4 C to precipitate DNA. Plates were centrifuged for 60 min at 3000 x g to pellet DNA and the PEG solution was removed. DNA pellets were washed twice with 300 μl of 70% ethanol, air-dried for 10 minutes and suspended in 100 μl of sterile water. DNA samples were quantitated using the Qubit dsDNA HS Assay Kit and diluted to 10 ng/μl.

### DNA amplification and 16S rRNA sequencing

The V4 region of the 16S rRNA gene was amplified in triplicate as previously described (Fujimura et al, 2016). Triplicate reactions were combined and purified using the SequalPrep Normalization Plate Kit (Invitrogen) according to manufacturer’s specifications. Purified amplicons were quantitated using the Qubit dsDNA HS Assay Kit and pooled at equimolar concentrations. The amplicon library was concentrated using the Agencourt AMPure XP system (Beckman-Coulter) quantitated using the KAPA Library Quantification Kit (KAPA Biosystems) and diluted to 2nM. Equimolar PhiX was added at 40% final volume to the amplicon library and sequenced on the Illumina NextSeq 500 Platform on a 153bp x 153bp sequencing run.

### Microbiota statistical analyses

Raw sequence data was converted from bcl to fastq format using bcl2fastq v2.16.0.10. Paired sequencing reads with a minimum overlap of 25bp were merged using FLASH v1.2.11. Index sequences were extracted from successfully merged reads and demultiplexed in the absence of quality filtering in QIIME (Quantitative Insights Into Microbial Ecology, v1.9.1), and reads with more than two expected errors were removed using USEARCH’s fastq filter (v7.0.1001). Remaining reads were de-replicated at 100% identity, clustered into operational taxonomic units (OTUs) at 97% sequence identity, filtered to remove chimeric sequences, and mapped back to OTUs using USEARCH v8.0.1623. Taxonomy was assigned using the Greengenes database (May 2013). OTUs detected in Negative Extraction Controls (NECs) were considered potential contaminants and filtered as follows: any known common contaminant OTU present in more than half of the NECs for this study was removed from all samples; the maximum read count for any OTU found in fewer than half of the NECs was subtracted from all samples; and any remaining OTU with a total read count less than 0.001% of the total read count across all samples was removed. Sequencing reads were rarefied to 47,594 reads for each sample as described previously (Fujimura et al., 2016).

Alpha-diversity indices were calculated in QIIME. Longitudinal comparisons between mouse genotypes were assessed by linear mixed-effects modeling, and per-month comparisons were evaluated by Welch’s t-test for binary variables and ANOVA with Tukey’s test for multiple comparisons. Results with a p-value of <0.05 were considered statistically significant.

Beta-diversity dissimilarity matrices (Bray-Curtis, Canberra, weighted and unweighted UniFrac distances) were generated in QIIME. Variables were assessed for their relationship to bacterial beta-diversity by permutational analysis of variance (PERMANOVA) using the Adonis function (vegan package) in the R environment; variables of p<0.05 were considered statistically significant.

Because data distributions differ between taxa, a “three model” (Poisson, negative-binomial, and zero-inflated negative-binomial models) approach was used to accommodate possible differences in data distributions and identify taxa exhibiting significant differences in relative abundance. The p-value associated with the model that minimized the Akaike information criterion value (AIC) (i.e., the best model fit for data distribution) was used for each taxon. Before applying the models, the OTU table was de-noised by removing taxa that were present in fewer than 25% of the samples. To adjust for multiple-testing, the Storey method to correct false-discovery in R (q-value package in Bioconductor) was calculated for each taxon; a q-value of <0.20 was considered significant.

## Supporting information

S1 Fig Between-group comparison of relative taxon abundance in A20dDC mice and control (Cre-) mice. Between-group comparison of taxon relative abundance in mice bearing A20 deficient DCs (A20dDC mice) and control (Cre-) mice. Relative abundance of the indicated taxons are shown for A20dDC and littermate control mice at the indicated ages. Color code for taxons shown below. Numbers of mice of each genotype sampled at each time point shown below each column.
(EPS)

S2 Fig Between-group comparison of relative taxon and genus abundance in adult A20dDC mice and control (Cre-) mice. Between-group comparison of relative taxon and genus abundance in adult mice (> 2months) bearing A20 deficient DCs (A20dDC mice) and control (Cre-) mice. Relative abundance of the indicated OTUs are shown for A20dDC and littermate control mice at the indicated ages. Color code for taxons shown below.
(EPS)

S1 Table Taxa significantly enriched in age matched A20dDC mice and control A20FL/FL (Cre-) mice. Tabular list of taxa enriched in A20dDC mice relative to control (Cre-) mice. OTUs that are relatively enriched in A20dDC mice are shaded grey; OTUs that are enriched in Cre- mice are shaded peach.
(XLSX)

S2 Table Taxa significantly enriched in young age matched A20dDC mice relative to control A20Fl/Fl (Cre-) mice. A20dDC mice versus A20Fl/Fl (Cre-) mice group’s comparison of mean relative abundance across repeated measures from 1, 2 or 4 week old littermate pups.
(XLSX)

S3 Table Taxa significantly enriched in 6 month old A20dDC RAG-1^-/-^ mice compared with control A20FL/FL (Cre-) RAG-1^-/-^ mice. Between groups comparison of mean relative abundance of microbial taxa in age matched lymphocyte deficient RAG-1-/- mice bearing either A20 deficient or A20 competent DCs. Taxa enriched in A20dDC RAG-1-/- mice are shown above. Taxa enriched in control (Cre-) mice are shown below.
(XLSX)
